# Pulmonary shunts in severe hepatosplenic schistosomiasis: Diagnosis by contrast echocardiography and their relationship with abdominal ultrasound findings

**DOI:** 10.1371/journal.pntd.0005417

**Published:** 2017-04-03

**Authors:** Liana Gonçalves-Macedo, Ana Lucia Coutinho Domingues, Edmundo Pessoa Lopes, Carlos Feitosa Luna, Vitor Gomes Mota, Mônica Moraes de Chaves Becker, Brivaldo Markman-Filho

**Affiliations:** 1 Graduate Program in Tropical Medicine, Universidade Federal de Pernambuco, Recife, Brazil; 2 Department of Clinical Medicine, Universidade Federal de Pernambuco, Recife, Brazil; 3 Center for Gastroenterology and Hepatology, Universidade Federal de Pernambuco, Recife, Brazil; 4 Laboratory of Quantitative Health Methods, Fundação Oswaldo Cruz (Fiocruz), Recife, Brazil; 5 Center for Cardiology and Echocardiography, Universidade Federal de Pernambuco, Recife, Brazil; George Washington University, UNITED STATES

## Abstract

**Background:**

Schistosomiasis is endemic to several parts of the world. Among the species that affect humans, *Schistosoma mansoni* is one of the most common causes of illness. In regions where schistosomiasis mansoni is endemic, reinfection is responsible for the emergence of hepatosplenic schistosomiasis (HSS) with portal hypertension in about 10% of infected individuals. Regardless of its etiology, portal hypertension may bring about the formation of arteriovenous fistulas and pulmonary vascular dilation, thus constituting a pulmonary shunt and its presence has been associated with the occurrence of neurological complications. The objective of this study was to identify pulmonary shunt using TTCE in patients with HSS and esophageal varices, and to compare the abdominal ultrasound and endoscopy findings among patients with and without pulmonary shunt.

**Methodology/Principal findings:**

In this case series, a total of 461 patients with schistosomiasis mansoni were prospectively evaluated using abdominal ultrasound and endoscopy and 71 presented with HSS with esophageal varices. Fifty seven patients remained in the final analysis. The mean age of the patients was 55 ± 14 years, and 65% were female. Pulmonary shunts were observed in 19 (33.3%) patients. On comparing the groups with and without pulmonary shunt, no significant differences were observed in relation to the abdominal ultrasound and endoscopic findings. When comparing the two subgroups with pulmonary shunts (grade 1 vs grades 2 and 3), it was observed that the subgroup with shunt grades 2 and 3 presented with a significantly higher frequency of an enlarged splenic vein diameter (>0.9 cm), and an advanced pattern of periportal hepatic fibrosis (P = 0.041 and P = 0.005, respectively). None of the patients with pulmonary shunts had severe neurological complications.

**Conclusions/Significance:**

Our findings suggest that in HSS with esophageal varices the pulmonary shunts may be present in higher grades and that in this condition it was associated with ultrasound findings compatible with advanced HSS.

## Author summary

Among the species of *Schistosoma* that infect humans *Schistosoma mansoni* is one of the most common causes of illness. In the areas where schistosomiasis mansoni is endemic, around 10% of infected individuals develop hepatosplenic schistosomiasis (HSS) with portal hypertension. Portal hypertension may promotes an imbalance in the hepatic production of vasoactive substances, which may act on the lungs promoting the formation of arteriovenous fistulas and pulmonary vascular dilation, a condition that is called a pulmonary shunt. When the pulmonary shunt is of higher grades, small thrombus or septic emboli that would normally be filtered through the pulmonary capillaries reach the left heart and the systemic circulation, which can lead to neurological complications. We found pulmonary shunts in patients with HSS and esophageal varices and we also found that patients with higher grades of pulmonary shunts presented with a significantly higher frequency of advanced periportal fibrosis and an enlarged splenic vein diameter. No neurological complications were observed. Our findings suggest that pulmonary shunts may be present in patients with HSS and esophageal varices. The abdominal ultrasound findings compatible with advanced HSS could be used as screening to investigate pulmonary shunt.

## Introduction

It is estimated that more than 200 million people in the world are infected with *Schistosoma* and more than 700 million remain at risk of infection, according to World Health Organiztion reports [1, 2]. Of the species of *Schistosoma* that infect humans, the two that most frequently cause illness are *Schistosoma haematobium*, found in Africa and the Middle East, and *Schistosoma mansoni*, found in parts of Africa, the Middle East, and the Americas [3]. In Brazil, *Schistosoma mansoni* infection affects all of the Northeastern states and parts of the North, South, Southeast, and Mid-West, and it is endemic in nine states [4, 5]. In these regions, reinfection is frequent and around 10% of the infected individuals develop severe forms of the disease, such as hepatosplenic schistosomiasis (HSS), while in hospital-based samples this percentage may reach higher values [6].

Generally, HSS presents with elevated pressure in the portal venous system, secondary to the deposition of *Schistosoma mansoni* eggs in the intrahepatic branches of the portal vein, which promotes an intense inflammatory process followed by periportal liver fibrosis (Symmers’ fibrosis), splenomegaly and gastroesophageal varices [7, 8].

In portal hypertension, regardless of the etiology, an imbalance in the hepatic production of angiogenic and vasoactive substances, coupled with a possible genetic predisposition, promotes the formation of pulmonary vascular dilations and pulmonary arteriovenous fistulas [9–11]. These may be responsible for the deviation of part of the blood from the pulmonary arteries directly into the pulmonary veins and the left chambers of the heart [12, 13]. This diverted blood reaches the systemic circulation without passing through the pulmonary capillaries, thus not undergoing the filtering function that the capillaries perform [12, 13]. This situation is characterized by non-physiologic pulmonary shunting, which may be responsible for complications due to paradoxical embolization, such as transient ischemic attack, ischemic stroke, brain abscess, or hemoptysis and hemothorax. The latter two being secondary to intrabronchial or intrapleural rupture of the fistulas [14–16]. This type of shunt differs from the so-called physiologic pulmonary shunt, which is not associated with any clinical complications, due to the deviation of a minimal volume of blood from the bronchial arteries directly into the pulmonary veins without passing through the pulmonary capillaries, in addition to the coronary venous blood drained directly into the left ventricle [13].

In schistosomiasis, the pathophysiological mechanism of non-physiological pulmonary shunt has yet to be elucidated. It is speculated herein that in patients with HSS and esophageal varices, pulmonary shunts may occur through the same mechanism observed in patients with portal hypertension secondary to other causes, with the formation of pulmonary vascular dilatations and arteriovenous fistulas. Another mechanism could be the migration of eggs from *Schistosoma mansoni* to the pulmonary vessels through portosystemic collateral vessels or through dilatation of the liver sinusoids [17–18]. This may result in local necrosis and the formation of pulmonary arteriovenous fistulas, as described in a single one case report [19].

Non-physiological pulmonary shunts are frequently investigated through transthoracic contrast echocardiography (TTCE) in portal hypertension due to hepatic cirrhosis as part of the assessment for liver transplantation [20, 21]. In hereditary hemorrhagic telangiectasia this is performed to determine the grade of intensity with the aim of preventing neurological complications, but it has been poorly studied in patients with schistosomiasis [16]. The objective of this study was to identify pulmonary shunts in patients presenting with HSS and esophageal varices using TTCE, and to compare the abdominal ultrasound and endoscopic findings between patients with and without pulmonary shunt. The secondary objective was to compare the abdominal ultrasound and endoscopic findings between the subgroups of patients with different shunt grades.

## Methods

### Ethics statement

The study was approved by the research ethics committee of the Federal University of Pernambuco Center for Health Sciences (Protocol no. 396/2010). All participating patients signed the informed consent forms.

### Patients

In this case series, patients previously diagnosed with *Schistosoma mansoni* infection (referred from primary health care services from all municipalities in the Pernambuco State) were prospectively screened for pulmonary shunts at a schistosomiasis mansoni outpatient clinic at a tertiary hospital (Hospital das Clínicas at the Universidade Federal de Pernambuco, HC-UFPE), between December 2010 and December 2012. Patients initially underwent an abdominal ultrasound in order to define the clinical form of schistosomiasis mansoni. Patients presenting with HSS were referred to undergo upper gastrointestinal endoscopy. The exclusion criteria for this phase of the study were: seropositivity for hepatitis B or C virus, alcohol over the limit of 60 g/day for males and 40 g/day for females during the previous six months, concomitant diagnosis of liver cirrhosis, splenectomy, previous diagnosis of ventricular dysfunction with a left ventricular ejection fraction ≤35% using echocardiogram and presence spirometry with a severe obstructive disorder or severe restrictive disorder. Patients with HSS and esophageal varices who met the inclusion criteria were referred for TTCE.

### Procedures

#### Abdominal ultrasound

**Fig 1:**
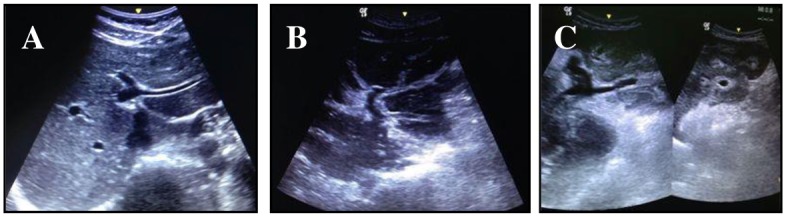
Abdominal ultrasound images of periportal fibrosis patterns. (A) Peripheral periportal thickening + central periportal thickening. (B) Advanced peripheral periportal thickening + central periportal thickening. (C) Very advanced central and peripheral periportal thickening.

Abdominal ultrasound was performed exclusively by one operator (ALCD-physician) specialized in diagnostic ultrasound for schistosomiasis mansoni. The equipment used was an Acuson X150 ultrasound system (Siemens Healthcare, Erlangen, Germany) with a 3.5 MHz transducer. The size of the spleen (its longitudinal diameter) was measured together with the cross-sectional diameters of the portal and splenic veins. We also determined whether collateral vessels were present. The pattern of periportal fibrosis was also categorized in accordance with the Niamey protocol [22]. The diagnosis of HSS was based on the finding of splenomegaly (spleen longitudinal diameter ≥13 cm) and the periportal fibrosis pattern (moderate, advanced, or very advanced) (Fig 1).

#### Upper gastrointestinal endoscopy

The upper gastrointestinal endoscopy was performed at the endoscopy department of the HC-UFPE with an EXERA endoscope, (Olympus Optical, Tokyo, Japan), following protocols to identify the presence and caliber of esophageal varices as well as portal hypertensive gastropathy [23].

#### TTCE

Patients with HSS and esophageal varices who met the inclusion criteria were referred for a TTCE (with a Philips Medical Systems, HDI 1500, Eindhoven, Netherlands), according to the protocol described below. A three-way catheter was inserted into a peripheral vein of the upper limb. Two 10 mL syringes were connected to the catheter, with one containing 9 mL of contrast material (0.9% saline solution). After the solution was shaken in the two syringes (ten times), it was injected into the catheter. The patient was considered positive for pulmonary shunting when microbubbles (contrast) were observed in the left atrium between the fourth and sixth cardiac cycles, after observing contrast in the right atrium through an apical four chamber view [24]. If there was left atrial opacification before the fourth cycle, the shunt was considered to be an intracardiac shunt, not a pulmonary shunt. To determine the grade of the pulmonary shunt, a semi quantitative classification according to the intensity of the left atrial opacification between the fourth and sixth cardiac cycles after right atrial opacification was used. The classification was as follows: grade 0, no microbubbles; grade 1, few microbubbles; grade 2, moderate amount of microbubbles, but not completely filling the left atrium; grade 3, many microbubbles completely filling the left atrium; grade 4, complete filling of the left atrium as densely as the right atrium [24]. Each test was performed twice for each patient. The second test was performed following the removal of all the microbubbles from all the chambers of the heart. A pulmonary shunt was considered to be present if any of the two tests were analyzed as positive. Two experts analyzed each test separately. If the image quality of the examinations proved inadequate for interpretation, they were considered inconclusive and therefore excluded.

Statistical analysis was performed with the Statistical Package for the Social Sciences, version 8.0 (SPSS Inc., Chicago, IL, USA). Continuous variables were expressed as mean ± standard deviation, and categorical variables were expressed as absolute and relative frequencies. To test the assumption of normality of the quantitative variables, the Kolmogorov-Smirnov test was used. The null hypothesis of normality was not rejected. As measures of association, we used the Student’s t-test for continuous variables and the Chi-square test, together with Fisher’s exact test, when indicated, for categorical variables. Values of p<0.05 were considered statistically significant. To compare the abdominal ultrasound and endoscopic parameters, cases without pulmonary shunts were used as controls. In a second stage, patients with pulmonary shunts were divided into two subgroups: those with grade 1 shunts and those with grade 2 or 3 shunts. The two subgroups were compared in terms of their abdominal ultrasound and endoscopic findings.

## Results

**Fig 2:**
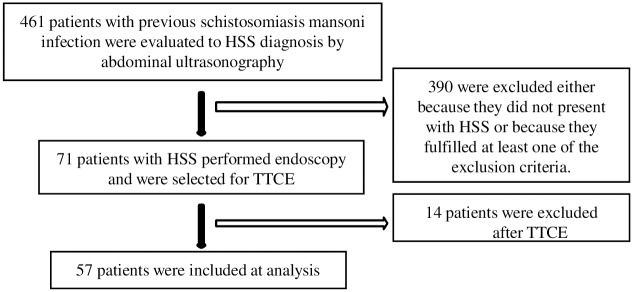
Flowchart of patient selection.

Four hundred and sixty one patients infected with schistosomiasis mansoni were initially assessed by abdominal ultrasonography. Three hundred and ninety were excluded either because they did not present with HSS or because they fulfilled at least one of the exclusion criteria. Seventy-one patients with HSS and esophageal varices were selected for TTCE. After performing TTCE, 14 patients were excluded: 4 because of a poor acoustic window, 1 for presenting with an atrial septal defect, 1 because of a patent foramen ovale, and 8 failed to complete the study. Therefore, the final study sample comprised 57 patients (Fig 2).

The mean age of the patients was 55 ± 14 years, and the majority (64.9%) were female (Table 1). Of the 57 patients, 35 (61.4%) presented with previous gastrointestinal bleeding due to ruptured esophageal varices, 28 (50.9%) with portal vein cross-sectional diameter > 1.2 cm, 31 (58.5%) with splenic vein cross-sectional diameter > 0.9 cm, 24 (42.1%) with collateral vessels, 40 (70.2%) with advanced or very advanced patterns of periportal hepatic fibrosis, 36 (63.2%) with medium or large caliber varices and 40 (70.2%) with portal hypertensive gastropathy (Table 1).

**Table 1:** Demographic and clinical characteristics and abdominal ultrasound and endoscopy parameters of patients with hepatosplenic schistosomiasis, with and without pulmonary shunts.

Characteristics	Total (n = 57)	Pulmonary shunt		P
		Yes (n = 19)	No (n = 38)	
Age (years), mean ± SD	55.02 ± 14.16	52 ± 19	57 ± 11	0.328
Gender, n (%)				
Male	20 (35.1)	5 (26.3)	15 (39.5)	
Female	37 (64.9)	14 (73.7)	23 (60.5)	0.389
Gastrointestinal bleeding, n (%)	35 (61.4)	11 (57.9)	24 (63.2)	0.777
Longitudinal diameter of the spleen (cm), mean ± SD	16.20 ± 2.58	16.14 ± 2.81	16.22 ± 2.49	0.911
Portal vein cross-sectional diameter (cm)^a^, mean ± SD	1.27 ± 0.32	1.28 ± 0.37	1.26 ± 0.29	0.785
≤ 1.2 cm, n (%)	27 (49.1)	10 (52.6)	17 (47.2)	
> 1.2 cm, n (%)	28 (50.9)	9 (47.4)	19 (52.8)	0.781
Splenic vein cross-sectional diameter (cm)^b^, mean ± SD	1.00 ± 0.32	1.06 ± 0.37	0.96 ± 0.29	0.271
≤ 0.9 cm, n (%)	22 (41.5)	6 (31.6)	16 (47.1)	
> 0.9 cm, n (%)	31 (58.5)	13 (68.4)	18 (52.9)	0.385
Collateral vessels, n (%)	24 (42.1)	7 (36.8)	17 (44.7)	0.777
Pattern of periportal fibrosis, n (%)				
Moderate	17 (29.8)	5 (26.3)	12 (31.6)	
Advanced/Very Advanced	40 (70.2)	14 (73.7)	26 (68.4)	0.766
Caliber of esophageal varices				
Small, n (%)	21 (36.8)	5 (26.3)	16 (42.1)	
Medium/large, n (%)	36 (63.2)	14 (73.7)	22 (57.9)	0.383
Portal hypertensive gastropathy, n (%)	40 (70.2)	16 (84.2)	24 (63.2)	0.132

^a^ No data available for two patients in the group without pulmonary shunt.

^b^ No data available for four patients in the group without pulmonary shunt.

SD, standard deviation

**Fig 3:**
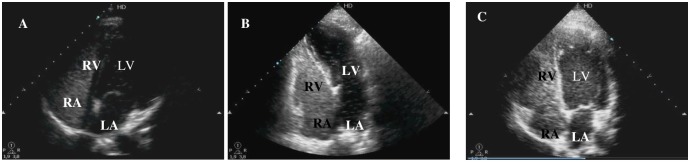
Transthoracic contrast echocardiography and grades of pulmonary shunt. (A) Grade 1. (B) Grade 2. (C) Grade 3. RV, right ventricle; RA, right atrium; LV, left ventricle; LA, left atrium.

Pulmonary shunts were identified by TTCE in 19 (33.3%) of the 57 patients, of whom, 8 (42.1%) were classified as grade 1, 8 (42.1%) as grade 2, and 3 (15.8%) as grade 3 (Fig 3). No pulmonary shunt grade 4 was recorded.

When the groups with and without pulmonary shunts were compared, no significant differences were observed in relation to the mean age, gender, previous gastrointestinal bleeding, or abdominal ultrasound and endoscopic findings (Table 1).

The abdominal ultrasound and endoscopic findings were compared between the two subgroups of patients with grade 1 pulmonary shunts versus those with grades 2 and 3. The mean values for the longitudinal diameter of the spleen, cross-sectional diameter of the portal vein, and cross-sectional diameter of the splenic vein were similar in both subgroups. There were also no significant differences in the presence of collateral vessels and in cross-sectional portal vein diameters >1.2 cm. There was a significantly higher frequency of splenic veins with a transversal diameter >0.9 cm and of an advanced or very advanced pattern of periportal liver fibrosis in the subgroup of patients with grade 2 and 3 pulmonary shunts when compared to the grade 1 subgroup (P = 0.041 and P = 0.005, respectively). There were no significant differences between the two subgroups in relation to the endoscopic parameters (Table 2). No neurological complications were recorded in the 19 patients with pulmonary shunts.

**Table 2:** Abdominal ultrasound and endoscopic parameters in patients with intrapulmonary shunts, by grade.

Parameter	Pulmonary shunt		p
	Grade 2 or 3 (n = 11)	Grade 1 (n = 8)	
Longitudinal diameter of the spleen (cm), mean ± SD	16.62 ± 2.86	15.48 ± 2.80	0.397
Portal vein cross-sectional diameter (cm), mean ± SD	1.32 ± 0.43	1.23 ± 0.28	0.588
≤ 1.2 cm, n (%)	5 (45.5)	5 (62.5)	
>1.2 cm, n (%)	6 (54.5)	3 (37.5)	0.650
Splenic vein cross-sectional diameter (cm), mean ± SD	1.10 ± 0.25	1.01 ± 0.52	0.619
≤ 0.9 cm, n (%)	1 (9.1)	5 (62.5)	
> 0.9 cm, n (%)	10 (90.9)	3 (37.5)	0.041
Collateral vessels, n (%)	5 (45.5)	2 (25.0)	0.633
Pattern of periportal fibrosis, n (%)			
Moderate	0 (0)	5 (62.5)	
Advanced/Very Advanced	11 (100)	3 (37.5)	0.005
Caliber of esophageal varices			
Small, n (%)	2 (18.2)	3 (37.5)	
Medium/large, n (%)	9 (81.8)	5 (62.5)	0.603
Portal hypertensive gastropathy, n (%)	10 (90.9)	6 (75.0)	0.546

## Discussion

In schistosomiasis, echocardiography is often performed as a screening test for the diagnosis of portopulmonary hypertension, yet TTCE is not currently included in the guidelines for assessment of these patients [25, 26]. In our study, we evaluated patients with HSS and esophageal varices, one of the significant findings was the diagnosis of pulmonary shunts in more than 30% of patients, and most of them (57.9%) classified with grade 2 and 3 shunts. However, at the time of collecting data on each patient there was no record of neurological complications that could be related to the diagnosis of a pulmonary shunt. Studies evaluating hereditary hemorrhagic telangiectasia patients have observed severe neurological complications in about 20% of patients with higher grades of pulmonary shunts [14, 27]. We emphasize that our case series study was a cross-sectional rather than a follow-up study, which may have impaired the observation of such complications. Although we have not reported severe neurological complications, the finding of grade 2 and 3 pulmonary shunts in HSS could indicate that consideration needs to be given to performing TTCE for diagnosing pulmonary shunts, in addition to screening for pulmonary hypertension.

Another prominent finding was the observation of a significantly higher frequency of abdominal ultrasound findings compatible with more advanced clinical forms of HSS in the subgroup of patients with higher grades of pulmonary shunts. It is noteworthy that the findings of our study differ from those of study using scintigraphy to diagnose pulmonary shunts in patients with HSS, in which the presence of pulmonary shunts was not associated with abdominal ultrasound parameters compatible with more severe forms of HSS [28]. Given that patients with more advanced periportal fibrosis progress to higher levels of portal pressure [29–31], our findings seem consistent with the current knowledge that associates portal hypertension with the presence of pulmonary shunts [32].

It is worth noting that abdominal ultrasound is not only used to diagnose the clinical form of schistosomiasis, but also to monitor progression of the disease in patients frequently exposed to water contaminated by *Schistosoma mansoni* [29, 31]. Thus, abdominal ultrasound could also be a useful screening tool for TTCE, especially in areas with limited resources that do not have easy access to TTCE. This could be initially indicated for patients with advanced periportal fibrosis and an enlarged splenic vein. These abdominal ultrasound findings are common in patients with esophageal varices who need to be referred to more advanced medical centers to undergo specialized treatment of portal hypertension, centers where TTCE may also be available.

**Fig 4:**
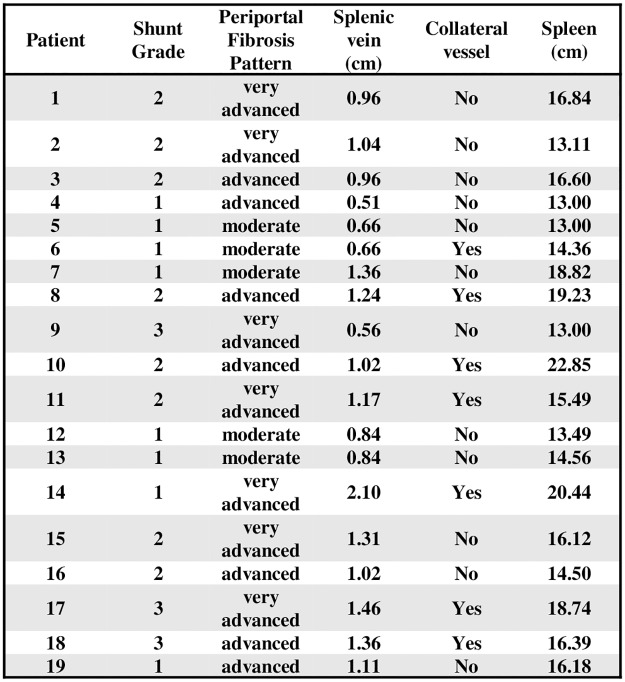
Main abdominal ultrasound findings in the 19 patients with pulmonary shunts.

Another point to be discussed in relation to the abdominal ultrasound findings of this study is the fact that no significant differences were observed in the frequency of portosystemic collateral vessels, neither when comparing patients with and without pulmonary shunts nor between the subgroups of patients with grade 1 pulmonary shunts versus grades 2 and 3. In fact, the presence of collateral vessels was observed in 7 of the 19 patients with pulmonary shunts, and in 5 of the 11 patients with grade 2 and 3 pulmonary shunts (Fig 4).

Indeed, this finding could be explained by the possibility that pulmonary shunts in patients with schistosomiasis may occur through a pathophysiological mechanism similar to that described in patients with portal hypertension secondary to other causes, and not by migration of parasite eggs through portosystemic collaterals. We also speculate that the formation of collateral vessels could work as a protective factor against the occurrence of pulmonary shunt, since it is a physiological mechanism that can reduce portal pressure levels in these patients.

We must also consider the limitations of this study. The fact that this is a case series, even though it had an analytical character because we included as controls patients without shunts and then the subgroup of grade 1 shunts, is a limitation. This study design allows us to establish hypotheses, but not to draw definitive conclusions on a causal association between the abdominal ultrasound variables (advanced periportal fibrosis and enlarged diameter of the splenic vein) and the presence of pulmonary shunts. These hypotheses may be tested in further studies with an analytical design.

Another limitation refers to patient selection, which was restricted to those with both HSS and esophageal varices. On the one hand, this inclusion criterion guaranteed the presence of portal hypertension in all patients. On the other, the presence of esophageal varices in all patients precluded the analysis of the association between the presence of esophageal varices and the pulmonary shunt. What we evaluated was whether the greater caliber of esophageal varices (medium/large) would be associated with the presence of the most severe grades of shunting (grades 2 and 3), which was not observed. Therefore, this inclusion criterion also limited the conclusions inferred by our findings to HSS with esophageal varices, and it would be not appropriate to extrapolate these results to all clinical presentations of schistosomiasis mansoni. A control group with HSS and no esophageal varices would also be needed to check for an association between the presence of esophageal varices and pulmonary shunts.

Finally, one further limitation may have been the fact that there was no previous study that defines the grade of left atrial opacification that may be considered a physiological pulmonary shunt through the semi quantitative technique of TTCE specifically for schistosomiasis mansoni. We attempted to minimize this fact by dividing the patients with pulmonary shunts into subgroups for analysis, where the subgroup with the least intense shunt, grade 1, was considered the control group.

In conclusion, our findings suggest that in HSS with esophageal varices pulmonary shunts may be present in higher grades. These findings may stimulate a discussion on performing TTCE in HSS with esophageal varices. In areas with limited resources, abdominal ultrasound findings compatible with advanced HSS could be used as screening parameters to perform TTCE. Follow-up studies in patients with HSS and pulmonary shunts may be useful to observe if neurological complications occur and how often they are present, in addition to observing whether they are associated with the presence of higher grades of pulmonary shunting.

## Supporting information

S1 Checklist STROBE statement. (DOC)
